# Optical and electrical characterizations of multifunctional silver phosphate glass and polymer-based optical fibers

**DOI:** 10.1038/srep43917

**Published:** 2017-03-03

**Authors:** Maxime Rioux, Yannick Ledemi, Steeve Morency, Elton Soares de Lima Filho, Younès Messaddeq

**Affiliations:** 1 Department of Chemistry, Université Laval, 1045, av. de la Médecine, Quebec, QC, G1V 0A6, Canada; 2 Center for Optics, Photonics and Lasers (COPL), Université Laval, 2375, rue de la Terrasse, Quebec, QC, G1V 0A6 Canada

## Abstract

In recent years, the fabrication of multifunctional fibers has expanded for multiple applications that require the transmission of both light and electricity. Fibers featuring these two properties are usually composed either of a single material that supports the different characteristics or of a combination of different materials. In this work, we fabricated (i) novel single-core step-index optical fibers made of electrically conductive AgI-AgPO_3_-WO_3_ glass and (ii) novel multimaterial fibers with different designs made of AgI-AgPO_3_-WO_3_ glass and optically transparent polycarbonate and poly (methyl methacrylate) polymers. The multifunctional fibers produced show light transmission over a wide range of wavelengths from 500 to 1000 nm for the single-core fibers and from 400 to 1000 nm for the multimaterial fibers. Furthermore, these fibers showed excellent electrical conductivity with values ranging between 10^−3^ and 10^−1^ S·cm^−1^ at room temperature within the range of AC frequencies from 1 Hz to 1 MHz. Multimodal taper-tipped fibre microprobes were then fabricated and were characterized. This advanced design could provide promising tools for *in vivo* electrophysiological experiments that require light delivery through an optical core in addition to neuronal activity recording.

Multimaterial and multifunctional fibers, which combine different properties and functions, have brought innovative technological solutions in the fields of smart textiles^1^,^2^,^3^, non-linear optics^4^,^5^,^6^, medicine^7^,^8^,^9^, optical transport^10^, fiber lasers^11^,^12^, chemical sensors and biosensors^13^,^14^. In 2015, an exhaustive and comprehensive review by Schmidt *et al*.^15^ reported the different multifunctional optical fibers developed in recent years for different applications in plasmonics^16^,^17^, optoelectronics^18^,^19^,^20^ and light generation^21^,^22^,^23^ for instance. These works illustrate the importance and the relevance of the efforts dedicated in the field of advanced fibers with the goal of offering novel approaches and solutions in many already existing and future applications. In particular, intensive research is currently under way in the field of multifunctional fibers for applications in electrophysiology^24^,^25^,^26^. However, all of the fibers fabricated to date for such applications are either not suitable for long-term measurements, not scalable, or have large diameters, ranging from 100 to 500 μm, strongly limiting or even impeding the local probing of few cells. For this targeted application, i.e. the high-spatial resolution recording of the neural activity of a small group of cells, fabricating fibers with small diameter like tapered fibers is of first importance. Developing novel multifunctional fibers that could be further stretched into small diameter tapers requires an appropriate combination of vitreous materials (including inorganic glasses and optical polymers) with compatible thermomechanical properties.

In the present study, we explored optical fibers based on electrically conductive AgI-AgPO_3_-WO_3_ (AAW) glasses previously investigated^27^ and standard low cost polymers–here polycarbonate (PC) and poly(methyl methacrylate) (PMMA). Glasses containing silver iodide (AgI) are well studied materials. They have been investigated for their ionic conductivity^28^,^29^,^30^, their structure^31^,^32^,^33^,^34^ and as electrochemically active materials^35^,^36^. The most well-known conductive glasses containing AgI are based on the AgI-AgPO_3_ system. It has been shown that they are among the best electrically conductive glassy materials at room temperature^28^, presenting an electrical conductivity as high as 10^−1^ S·cm^−1^. AgI is used as the conductor medium and silver phosphate (AgPO_3_) as the glassy matrix that allows the dissolution of high concentrations of silver ions without crystallization. However, because of the metaphosphate matrix, these glasses have poor chemical resistance against moisture, and will degrade by different hydration and hydrolysis reactions^37^. To improve their chemical durability with water, heavy metallic atoms in oxide form can be added to the phosphate matrix, like tungsten oxide WO_3_ for example^38^,^39^,^40^. The heavy atoms interact with the non-bridging oxygens of the matrix, which in turn become unavailable to interact with water. In our case, the effect of WO_3_ on the chemical resistance of the AAW glasses has been proven effective to stabilize the glassy materials against humidity^27^. Practical utilization can be thus envisaged for these AAW glasses to take full advantage of their unique capability to combine various properties in a single material: they conduct electricity, are optically transparent, are stretchable into fibers and depending on their composition, are also durable against water. Another advantage of these materials for the targeted application is their ionic nature. Since the conduction of electricity is assured by ions, there is practically no photoelectric effect coming from the material, preventing therefore the formation of electrical artifacts that can appear in applications requiring metallic electrodes, as in electrophysiological recordings^41^. In addition, the utilization of an electroconductive glass whose electrical resistance is in the order of the megaohm permits a better discrimination of the probed cells, which is hardly achieved with highly conductive electrical-optical fibers. All these particular features combined in one single material make the AAW glasses promising candidates for the development of multifunctional fibers for electrophysiology.

Here, we present the fabrication and characterizations of different designs of optical fibers, including taper-tipped fibers. The first optical fiber investigated is made of AAW glasses with an acrylic cladding that can be used for the fabrication of electrically conductive fibers that transmit light in the near infrared. The second one relies on two materials and combines the electrical properties of the AAW glasses with the optical transparency of the polycarbonate (PC) in the visible spectral range. The characterization of these fibers includes light attenuation measurements between 400 nm–1000 nm and the measurement of electrical properties at AC frequencies from 1Hz to 1MHz, at temperatures between 25 °C and 75 °C.

## Results and Discussion

### Fiber designs

**Figure 1:**
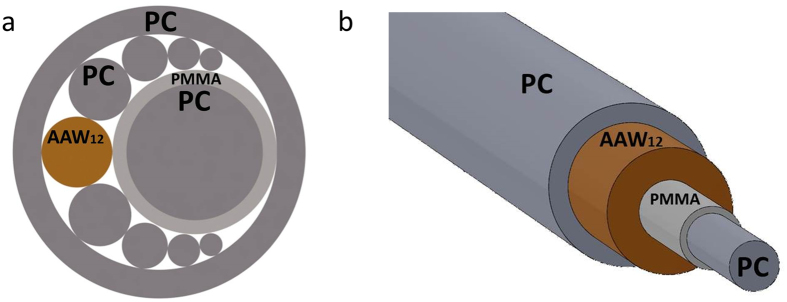
Preform cross-section illustrations of: (**a**) the two circular-core fiber, and (**b**) the ring-core fiber. Material labels: PC: polycarbonate, PMMA: poly(methyl methacrylate), AAW_12_: 45AgI-43AgPO_3_-12WO_3_ glass (mol%).

**Figure 2:**
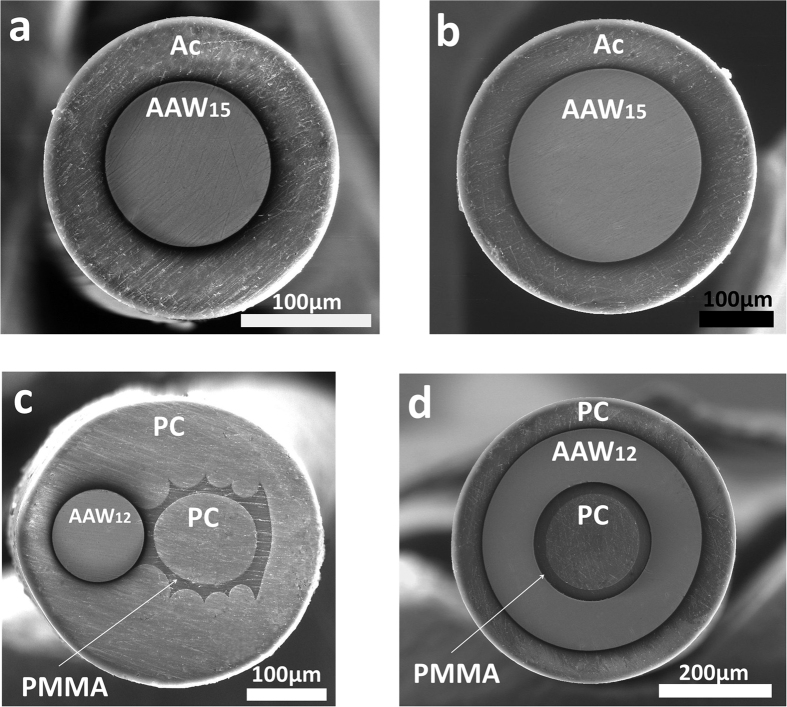
Cross-section SEM images of: (**a**,**b**) 125 μm and 250 μm diameter single-core AAW_15_ glass fibers with acrylic coating (Ac); (**c**) two circular-core fiber with 125 μm diameter AAW_12_ glass and 140 μm diameter PC cores and (**d**) ring-core fiber with a PC cladding ring of 45 μm thickness, an electrically conductive AAW_12_ glass ring of 90 μm thickness, a PMMA ring of 25 μm thickness, and a PC core of 155 μm diameter. Glass labels: AAW_12_: 45AgI-43-AgPO_3_-12WO_3_, AAW_15_: 45AgI-40-AgPO_3_-15WO_3_.

As depicted in the following sections, fibers with single-core and dual-core cross-section geometries were fabricated. In the single-core fiber design, the cladding is made of standard acrylic polymer, and both optical and electrical transports take place within the AAW glass core. It is worth mentioning that the acrylic polymer cladding here plays two roles: (i) as a protective fiber coating and (ii) as a low index optical medium to enable the total internal reflection phenomenon required for light guiding. The fabrication method used to produce the AAW fibers is very simple: it indeed consists in drawing only one single material –the 45AgI-40AgPO_3_-15WO_3_ glass (mol%, labelled AAW_15_)–from the preform while an acrylic polymer coating is applied and UV-cured along the same process. For the dual-core fibers, the optical and electrical functionalities are decoupled into two separate channels to facilitate their respective connections for practical use and electrical/optical probing. Two designs of dual-core fibers with different schemes for electrical and optical connections were considered in this work. The first one consists in a fiber with two circular cores with an offset from the fiber’s central axis, wherein the electrically conductive core is made of 45AgI-43AgPO_3_-12WO_3_ glass (mol%, labelled AAW_12_), while the light guiding core is made of commercial transparent polycarbonate (PC) and poly(methyl methacrylate) (PMMA) polymers for the core/cladding configuration, respectively. The second dual-core fiber design consists in a fiber having two concentric cores: the inner core being made of PC polymer for light guiding, while the outer core with a ring shape is made of our electrically conductive AAW_12_ glass. The general layout of the two circular-core and ring-core preforms is presented in Fig. 1 and the scanning electron microscope (SEM) images of the resulting fibers are presented in Fig. 2. It is important to notice that all fiber geometrical designs rely on large, multimode optical and electrical cores covering a substantial cross-sectional area to maximize the electrical conductivity and enlarge the optical collection area.

### Material selection

**Figure 3:**
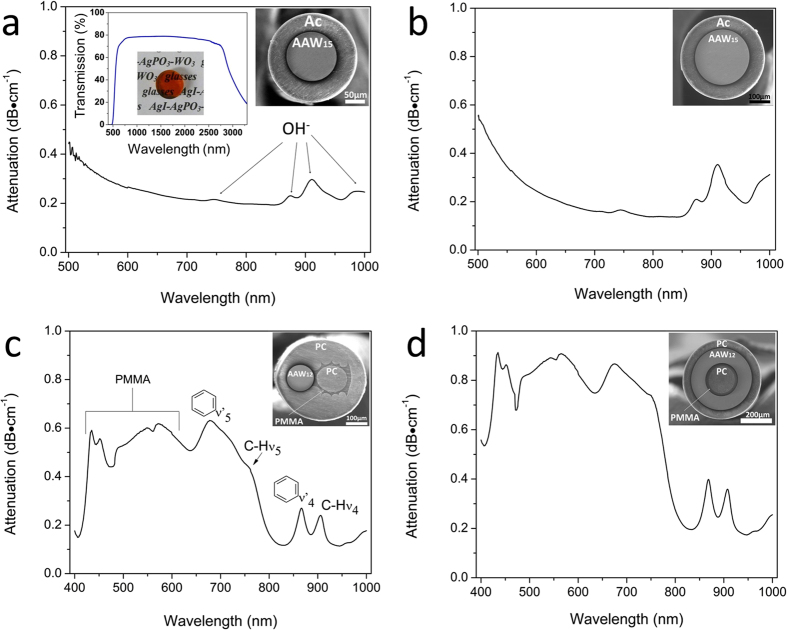
Attenuation spectra of: (**a**,**b**) the 125 μm and 250 μm diameter single-core AAW_15_ glass fibers with acrylic coating (Ac); (**c**) the two circular-core fiber with 125 μm diameter AAW_12_ glass and 140 μm diameter PC cores and; (**d**) the ring-core fiber with a PC cladding ring of 45 μm thickness, an electrically conductive AAW_12_ glass ring of 90 μm thickness, a PMMA ring of 25 μm thickness, and a PC core of 155 μm diameter. Corresponding SEM cross-section images of the fibers are shown in inset. Glass labels: AAW_12_: 45AgI-43-AgPO_3_-12WO_3_, AAW_15_: 45AgI-40-AgPO_3_-15WO_3_.

For the single-core glass fibers, the AAW_15_ glass composition was selected since it provides the best compromise amongst previously studied glasses according to optical transparency, electrical conductivity and chemical durability criteria^27^. That composition exhibits high thermal stability against crystallization with no apparent crystallization peak up to 600 °C on its DSC thermogram. Such stability was ascribed to the increase of the glass crystallization activation energy when silver iodide AgI is incorporated into glassy materials^27^,^42^,^43^. This glass characteristic is highly desirable for fiber drawing capability. Moreover, the AAW_15_ glass is transparent between 500 nm and 2800 nm (Fig. 3a) with a transmission maximum of ~80% between 750 nm and 2500 nm for a 3 mm-thick bulk sample. The optical properties of the glass are the result of the formation of tungsten iodide species (W_y_I_x_) that can be formed in the glassy matrix and results in a reddish colored glass^27^,^44^,^45^. The chemical resistance of that composition against water has been previously evaluated by the soaking method in deionized water at 60 °C for 48 hours^27^. The corresponding weight loss per surface unit equals to (0.4 ± 0.04) mg/cm^2^. Thus, we expect these fibers to be durable enough for practical use in ambient conditions and for the targeted application. The cladding material used for the single-core fibers is acrylic. It is a mass-produced commercial polymer that can be used on the surface of fibers to improve its mechanical resistance. Furthermore, acrylic has a low refractive index *n* varying from 1.50 down to 1.48 in the wavelength range 435 nm–1050 nm. By taking into account the high refractive index of the AAW_15_ glass core in the visible range (*n* ~ 2.0) compared to that of the acrylic cladding (*n* ~ 1.5), a large numerical aperture (i.e. NA > 1, calculated with 
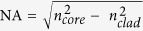
) is obtained for these single-core fibers (Supporting Information in Fig. S1)^46^.

For the dual-core glass-polymer composite fibers, PC polymer was used as cladding material to provide biocompatibility^47^,^48^,^49^ as well as protection against the environment for the AgI-AgPO_3_-WO_3_ glass. Then, while electrical conduction is assured by the AgI-AgPO_3_-WO_3_ glass core, optical signal transport is enabled by a PC/PMMA core/cladding configuration, as shown in Fig. 1c,d. PC polymer was used as an optical core to transmit light since it provides high transparency from 400 to 1000 nm^50^. The step index between PC and PMMA materials that ensure light guidance by total internal reflection yields numerical apertures (NA) between 0.50–0.58 in the 450–1000 nm wavelength range. Such large NAs allow for relatively strong light confinement in the PC core and a large acceptance cone of light (Supporting Information in Fig. S2)^51^. Moreover, the polymer and glass materials utilized in these composite fibers should have similar thermal and thermomechanical properties (glass transition temperature *T*_g_ and softening temperature *T*_soft_) since they are co-heated and co-stretched from a single pre-assembled preform. Therefore, the materials must possess similar viscosities and viscoelastic behaviours at the drawing temperature^26^. To fulfil those requirements, the glass nominal composition was adjusted to 45AgI-43AgPO_3_-12WO_3_ (AAW_12_). In addition to its electrical conductivity properties and its moisture resistance, which are similar to those of the 45AgI-40AgPO_3_-15WO_3_ glass (AAW_15_) composition in the single-core fiber, the AAW_12_ glass exhibits an excellent fiber formability, without any crystallization during fiber drawing, and thermal compatibility (*T*_*g*_ = 175 °C, *T*_*soft*_ = 200 °C) with commercial-grade polycarbonate (PC, *T*_*g*_ = 147 °C, *T*_*soft*_ = 155 °C) and polymethylmethacrylate (PMMA, *T*_*g*_ = 105 °C, *T*_*soft*_ = 160 °C).

### Optical characterization

#### Single-core fiber

The fiber’s optical attenuation was recorded using the cut-back method on 60 cm and 40 cm of fiber length. The optical attenuation spectra obtained from 125 and 250 μm diameter single-core fibers are presented in Fig. 3a,b, respectively. The transmission spectrum of the AAW_15_ glass bulk is shown in Fig. 3a in inset. One can observe a similar optical attenuation from both the glass and the fibers in the short wavelength band edge. A minimum attenuation of 0.15 to 0.20 dB⋅cm^−1^ was measured in the near-infrared wavelength range near 800 nm. The different absorption bands centred at 745 nm, 875 nm, 910 nm and 985 nm for the 125 and 250 μm fibers are attributed to the presence of hydroxyl (OH^−^) groups related to the presence of water (in the glass and/or the polymer cladding) which is usually observed in non-purified silica optical fibers^52^,^53^. Guiding of residual light in the polymer cladding may be also considered. Below 500 nm, the conductive glass absorbs practically all the light and its attenuation spectrum cannot be measured. Incidentally, the wavelength range for an effective practical use of these single core fibers is located in the near-infrared. At this point, none of the conductive glasses could offer a good compromise between optical transmission in the blue region, electrical conductivity and durability. Thus, the fabrication of a composite fiber with a PC optical core transmitting blue light was investigated and is described in the following sections.

#### Two circular-core and ring-core fibers

The AAW_12_ glass and PC core diameters were respectively measured to be 125 and 140 μm after fiber drawing, as shown in the SEM picture of Fig. 1c. The optical attenuation of the two circular-core fiber measured in the PC core is reported in Fig. 3c. In the visible region, the optical attenuation was measured to be ~0.6 dB⋅cm^−1^ and is dictated by the optical transparency of the polycarbonate material. In the near-infrared region, the optical attenuation dropped down to ~0.2 dB⋅cm^−1^. Between 400 and 650 nm, the different absorption bands are attributed to the high harmonic vibrations from the stretching vibrations of C-H groups and intrinsic electronic transitions of PMMA^54^. Between 650 and 1000 nm, the bands are attributed to the stretching vibrations of aromatic groups and the fundamental aliphatic C-H bending vibrations of PC^55^. The absorption bands between 400 and 600 nm are related to PMMA and suggest losses in the optical cladding mainly due to the intrinsic carbon-hydrogen vibrations. Furthermore, losses may be induced by scattering from existing micro-bubbles and voids as well as by geometrical variation of the optical core diameter along the axis of the fiber. It can be seen from Fig. 3 that these fibers may be employed for transmitting light at wavelengths around 405 nm, 445 nm, 475 nm, 640 nm, 830 nm, 885 nm, 945 nm. This spectrum profile is observed for all PC/PMMA core cladding fibers presented in this work.

The ring-core fiber design consisted in one inner light guiding core made of polycarbonate polymer, surrounded by one outer ring of electrically conductive AAW_12_ glass. The ring-core fiber optical attenuation is reported in Fig. 3d. In the visible region, the optical attenuation was measured at ~0.6 dB⋅cm^−1^ while in the near-infrared region, the optical attenuation also dropped down to ~0.2 dB⋅cm^−1^. These optical attenuation results were similar to those obtained for the two circular-core fiber (Fig. 3c).

#### Electrical characterizations

**Figure 4:**
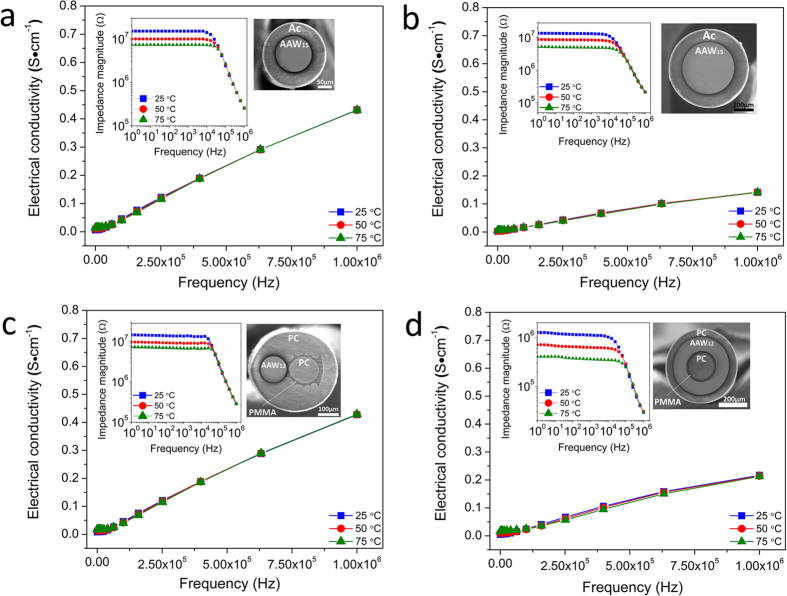
Electrical conductivity with the AC impedance magnitude spectra in inset as a function of the frequency at different temperatures for: (**a**,**b**) the 125 μm and 250 μm diameter single-core AAW_15_ glass fibers with acrylic coating (Ac); (**c**) the dual-core fiber with 125 μm diameter AAW_12_ glass and 140 μm diameter PC cores and (**d**) the ring-core fiber with a PC cladding ring of 45 μm thickness, an electrically conductive AAW_12_ glass ring of 90 μm thickness, a PMMA ring of 25 μm thickness, and a PC core of 155 μm diameter. Fiber length: 15 cm. Glass labels: AAW_12_: 45AgI-43-AgPO_3_-12WO_3_, AAW_15_: 45AgI-40-AgPO_3_-15WO_3_.

Impedance measurements were performed as a function of temperature, from 25 °C up to 75 °C within the range of AC frequencies from 1 Hz up to 1MHz for all the 15 cm-long fibers. As expected, the fiber impedance is lower for the 250 μm glass core compared to the 125 μm glass core since its cross-section is larger, as depicted in inset on Fig. 4a,b. However, the electrical conductivity of the small 125 μm core fiber is higher due to the higher relative contribution of surface conductivity at the glass-polymer interface^27^. Indeed, an increase of the electrical conductivity can be observed by increasing the surface/volume ratio as reported in Table 1.

From Fig. 4a,b, ~5 MΩ impedance is expected at low frequencies at 25 °C with 5 cm-long single-core fibers of 125 μm and 250 μm core diameter. At low frequencies, i.e. 1Hz-10 kHz, an impedance plateau is observed. Our hypothesis to explain the origin of the latter is as follows: at high frequencies the temperature is sufficiently high to provide the silver ions the thermal energy required to cross the energy barriers for the conduction of electricity. As a result, the conductivity is strongly dependent on the frequency of the AC current. Then, as the frequency decreases down to about 10 kHz, the temperature becomes too low for the ions to move from one site to another and the impedance magnitude progresses very slowly while becoming independent of the frequency.

**Figure 5:**
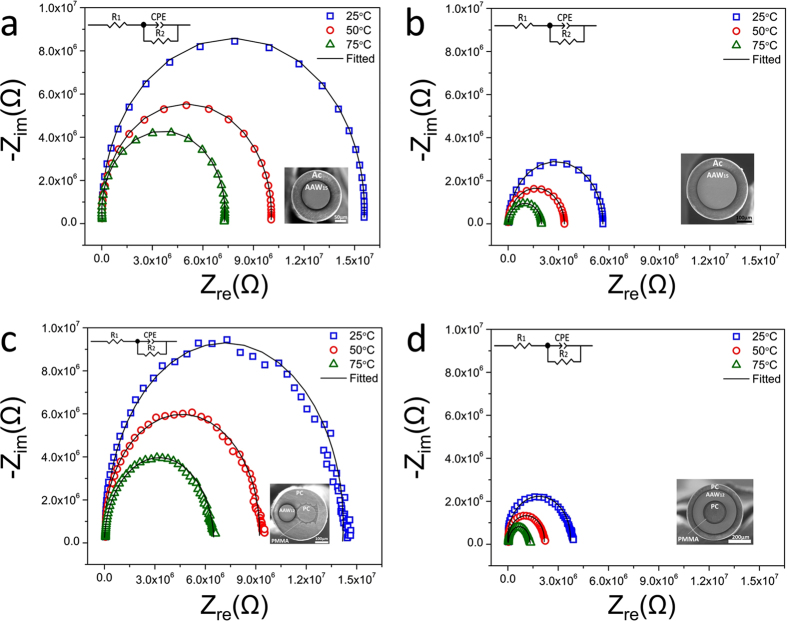
Nyquist spectra for: (**a**,**b**) the 125 μm and 250 μm diameter single-core AAW_15_ glass fibers with acrylic coating (Ac); (**c**) the dual-core fiber with 125 μm AAW_12_ glass and 140 μm diameter PC cores and (**d**) the ring-core fiber with a PC cladding ring of 45 μm thickness, an electrically conductive AAW_12_ glass ring of 90 μm thickness, a PMMA ring of 25 μm thickness, and a PC core of 155 μm diameter. Fiber length: 15 cm. Glass labels: AAW_12_: 45AgI-43-AgPO_3_-12WO_3_, AAW_15_: 45AgI-40-AgPO_3_-15WO_3_.

The profile of the impedance curves is typical of a parallel resistance-capacitance (RC) circuit, which is attributed to the bulk resistance (R_1_) and the imperfect capacitance (constant phase element, CPE) of the ion conducting glass fiber. Since we have a RC circuit, we can assign the values resulting from that plateau to the sum of the bulk resistance (R_1_) and the charge transfer resistance (R_2_) (see discussion of Fig. 5 for more details). Moreover, the values of impedance and conductivity at three different temperatures tend to coincide at high frequency. This behaviour has been observed and discussed extensively in different complex ceramics (e.g. BaBi_4_Ti_4_O_15_, KCa_2_Nb_5_O_15_ and Li_2_Pb_2_Sm_2_W_2_Ti_4_TaO_30_) and may be the result to the space charges releasing^56^,^57^,^58^,^59^. That phenomenon corresponds to an excess of electric charges in a specific volume, resulting in a reduction in the energy barrier of the material with the rise in temperature and frequencies. It may also be a major factor for the increase of AC conductivity. At low frequencies, the impedance decreases with rise in temperature, because of the faster ion dynamics.

The electrical conductivity and impedance of the dual-core and ring-core fibers are presented in Fig. 4c,d. A~5 MΩ impedance is also expected at low frequencies at 25 °C with a 5 cm-long two circular-core fiber, as obtained for the 125 and 250 μm single-core fibers. Such result was anticipated since the glass composition and the electrical core diameter are similar in both two circular-core and single-core fibers. As for the ring-core design, an impedance of ~1.5 MΩ is expected with a 5 cm-long ring-core fiber. As explained above, we attribute the lower electrical conductivity of the ring-core fiber compared to the two circular-core fiber to its lower surface/volume ratio (i.e. lower surface conductivity contribution, see Table 1).

The thermal activation energy at low frequencies, between 1 Hz–10 kHz, for the single-core and the two circular-core fibers was calculated to be ~0.15 eV whereas a value of ~0.2 eV was obtained for the ring-core fiber, using the Arrhenius equation:


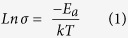


where *σ* is the conductivity in S·m^−1^, *E*_a_ the activation energy in eV, *k* the Boltzmann constant and *T* the temperature in Kelvin. These values are consistent with those obtained from bulk glasses in our previous study where an ionic conduction mechanism was demonstrated. These thermal activation energies are indeed well below the ~2.5 eV electronic transition energies related to the optical band edge near 500 nm wavelength for the AAW bulk glasses^27^. The Arrhenius plot and the profile of the impedance curves suggest the presence of an ionic conduction dominated by a hopping process.

The impedance complex Nyquist spectra displayed in Fig. 5 have been measured at three different temperatures as a function of the frequency. The Nyquist spectra allow determining the electrical behaviour of electro-conductive materials. For each fiber geometry, the equivalent circuit model corresponds to a simple resistance-capacitance parallel circuit. The working principle of this circuit can be explained as follows: at high frequencies, the imperfect capacitance (CPE) conducts easily, leaving in large part the resistance effect of the conduction series resistance (R_1_). When the frequency decreases, the electrical conduction contribution of the CPE diminishes and the response due to the charge transfer resistance increases (R_2_). As the frequency approaches zero, the capacitor does not conduct anymore and the impedance magnitude is a function of only R_1_ and R_2_. The Nyquist spectra reveal that there is an increase of the fiber’s capacitance with the temperature. This capacitive effect displacement corresponds to a higher dissociation of ions in the glass, increasing hence the concentration of ions at the double electrode/glass interfaces. This behaviour has also been observed in bulk glasses^27^.

#### Taper-tipped fibers

**Figure 6:**
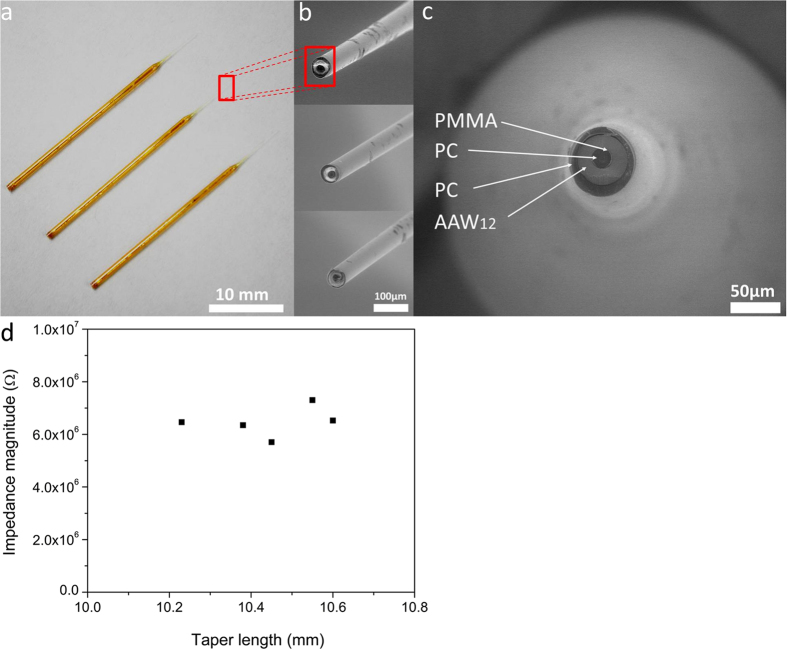
(**a**) Optical photograph of the multimaterial ring-core microprobe taper comprising AAW_12_ glass, PC, and PMMA materials; (**b**) scanning electron micrograph of the uncleaved taper part of the fibers; (**c**) magnification of a cleaved tapered-tip fiber and (**d**) measured AC electrical impedance distribution of 5 ring-core tapers, as a function of tip length, voltage: 1 V, frequency: 1 kHz. Glass labels: AAW_12_: 45AgI-43-AgPO_3_-12WO_3_.

Tapered fibers were produced in order to attain the high spatial resolution generally required in electrophysiological experiments and to minimize the damaging of biological tissues during *in vivo* cell probing^24^,^60^,^61^. The excellent co-drawing ability of the composite preform and fibers also is a clear advantage if compared to other multimaterial fibers for which no taper has been successfully produced for electrophysiology, to the best of our knowledge. The ring-core fiber was selected for conducting the taper-tipped fabrication. Its design indeed allows simple optical connection by using a standard fiber optics holder while facilitating electrical connection for electrophysiological applications. Moreover, the use of a PC optical core is preferred to the single-core fibers because of its better transmission in the visible spectrum, which is a critical parameter in electrophysiology. Indeed, blue light is often used for the excitation of the nervous cells, which have been optogenetically modified by the Channelrhodopsin-2 (ChR2) protein, a blue light-gated ion channel^62^,^63^. The ring-core fiber employed for these experiments was larger in size than those described in the previous section for better bending stiffness, better electrical connection and for efficient light delivery. More specifically, the fibers used to produce the taper-tipped fiber consisted in 1 mm external diameter fibers with a 260 μm diameter inner optical core made of PC polymer cladded by PMMA, surrounded with a 200 μm thick ring of electrically conducting AAW_12_ glass. The fiber tip was pulled thermally to form a taper, yielding a reduction of cross-sectional features by up to 20 times with a taper length of (10 ± 0.6) mm, an optical core of (25 ± 5) μm diameter at the tip and an overall length of 60 mm. The length of the taper was set to 10 mm since it is the standard length in many electrophysiological applications. Figure 6a–c show macroscopic and microscopic views of the thermally tapered ring-core microprobes. The AC electrical impedances were measured as a function of the tip length, as presented in Fig 6d. The five selected taper-tipped fibers exhibited AC electrical impedance of (5.7 ± 1.8) MΩ, which is a typical range of operation in commonly used *in vivo* electrophysiological recordings^24^. This result is consistent with the expected AC impedance of the ring-core fiber given above which is situated around 1.5 MΩ for a 5 cm-long fiber. This suggests that the taper itself might contribute measurably to the overall electrical impedance of the microprobe since the size of the fiber end was considerably reduced.

## Conclusion

The objective of this work was to fabricate and characterize different multifunctional fibers comprising AgI-AgPO_3_-WO_3_ glass and PC/PMMA polymers for concurrent electrical conduction and light transmission. To this end, various multimaterial optical fibers of different designs combining both electrical and optical signal transport capability were produced and characterized. We demonstrated that the single-core AgI-AgPO_3_-WO_3_ fibers and the dual-core AgI-AgPO_3_-WO_3_/polymer fibers can provide optical transmission in the near-infrared ranges (i.e. 700–1000 nm) and the visible respectively, with attenuation in the order of 10^−1^ dB·cm^−1^. At low frequency, the single core and two circular-core fibers showed electrical resistance around 5 MΩ for 5 cm-long fibers at 25 °C. The ring-core fiber showed an electrical resistance corresponding to 1.5 MΩ for a 5 cm-long fiber in the same conditions. The electrical conductivity of each fiber tends to increase when increasing the surface/volume ratio, which indicates that the surface contributes to the overall conductivity of the fibers. The thermal activation energy of the fibers at low frequencies calculated from the Arrhenius equation is in the order of 10^−1^ eV and is indicative of an ionic conductivity mechanism. The ionic nature of the fibers’ conductivity is also supported by the decrease of the electrical resistance with the temperature. Thermal fiber tapering of the ring-core fiber was successfully achieved, resulting in thin tapers with an optical core of (25 ± 5) μm diameter that can be further employed for minimally invasive deep brain structure access for light-mediated neuronal interrogation. The design is easily amenable to microstructuring approaches, which may prove instrumental for diverse applications. We believe that this new multifunctional fiber could be a new tool for applications that require the simultaneous conduction of electricity and the transmission of light such as the recording and light-mediated manipulation of neuronal activity, which could be of interest for a broad use by the neuroscience community.

## Methods

### Bulk glass synthesis and optical fibers fabrication

#### Materials

Tungsten (VI) oxide WO_3_ powder (99.9%) and ammonium phosphate monobasic NH_4_H_2_PO_4_ (≥98.5%, ReagentPlus grade) were both obtained from Sigma Aldrich. Silver iodide AgI (99.9%) and silver nitrate AgNO_3_ (99.9%, ACS grade) were both obtained from Alfa Aesar. All reagents were used as received. The polycarbonate (PC) and polymethylmethacrylate (PMMA) were both obtained from McMaster-Carr.

### Fabrication of the 45AgI-(55-x)AgPO_3_-xWO_3_ glass preforms with x = 12, 15 mol%

The 45AgI-(55-*x*)AgPO_3_-*x*WO_3_ glass preforms with *x* = 12 and 15 mol%, were synthesized according to the method previously described^27^. Briefly, the silver metaphosphate (AgPO_3_) has been first synthesized using a mixture of silver nitrate (AgNO_3_) and ammonium phosphate monobasic (NH_4_H_2_PO_4_) in a mass ratio AgNO_3_/NH_4_H_2_PO_4_ of 1.48. The mixture was then heated at 350 °C during 24 h and cast into a stainless steel mould at room temperature. The silver iodide (AgI) and the tungsten oxide (WO_3_) were then added to the as-prepared AgPO_3_ at a temperature of 1000 °C during 10 minutes in a silica crucible. The high temperature glass liquid was then cast in a preheated brass mould with a diameter of 12.0 mm and a length of 12.0 cm. The preforms were annealed at a temperature of 175 °C overnight to remove any residual stress. The glass preforms were then polished using silicon carbide (SiC) polishing papers with decreasing grit size (400 and 800).

### Fabrication of the single-core 125 and 250 μm 45AgI-40AgPO_3_-15WO_3_ (AAW_15_) optical fibers

To fabricate the AAW_15_ optical fibers with acrylic cladding, cylindrical glass preforms of 70 mm in length and 10 mm in diameter were prepared using a preheated cylindrical brass mould. The glass preforms were drawn into fibers in a 7-meter optical fiber drawing tower set with controlled diameters of (125 and 250 ± 5) μm at a temperature of 300 °C. A UV-cured acrylic resin coating (DeSolite DS-2015) was applied to the fiber surface for mechanical resistance improvement.

### Fabrication of the dual-core fiber

A 45AgI-43AgPO_3_-12WO_3_ glass (AAW_12_) rod was stretched down from 12 mm diameter to 5.3 mm diameter in order to adjust its size with respect to the 20 mm diameter PMMA tubing that formed the outer preform cladding. The PC rods and PMMA tube were annealed at 120 °C during 24 h under a nitrogen flow to remove residual water in order to prevent the formation of bubbles during fiber drawing. These materials were then stacked into a preform with an apollonian gasket arrangement that maximized tube filling following the general cross-sectional layout illustrated in Fig. 4a. Then, the preform was drawn into a fiber at a temperature of 220 °C with controlled diameters of (125 and 140 ± 5) μm for the conductive glass core and the PC core, respectively.

### Fabrication of the 45AgI-43AgPO_3_-12WO_3_ (AAW_12_) glass tube

The AAW_12_ glass tube was prepared by using the rotational casting method^64^. Briefly, the glass melt was cast at high temperature in a brass mould with a diameter of 12 mm and a length of 120 mm. The mould, which is in vertical position for the casting, is embedded in a furnace and coupled to a rotational system. Once the batch is poured, the mould is put in horizontal position and a subsequent rotation of 600 rpm is applied. A tube is then formed while the glass is solidifying under centrifugation, giving an internal diameter equal to (8 ± 0.5) mm. Finally, the tube is annealed overnight and polished following the same procedure described above.

### Fabrication of the ring-core fiber

The preheated PMMA tube and the PC rod were stretched to dimensions that fit with the internal diameter of the above prepared glass tube. The fabrication of the composite preform was done by inserting the PMMA tube and the PC rod inside the conductive glass tube (Fig. 5a). The preform was then drawn into a fiber at a temperature of 220 °C, at a rate that leads to a fiber cross-section with a PC ring of (45 ± 5) μm thickness, a conductive glass ring of (90 ± 5) μm thickness, a PMMA ring of (25 ± 5) μm thickness, and a PC core of (155 ± 5) μm diameter.

### Fabrication of the taper-tip fiber

The taper-tip microprobe was fabricated using the same procedure than the ring-core design but with a 1 mm external diameter for increasing bending stiffness and for efficient light delivery through the tip of the fiber. To achieve single-cell resolution with the microprobe, the fibers have been pulled to micron-scale tip sizes using a vertical puller (Narishige, PP-83) equipped with a heating element as commonly used in electrophysiological laboratories, yielding a reduction of cross-sectional features by up to 20 times with a taper length of (10 ± 0.6) mm and an optical core of (25 ± 5) μm diameter. All the fibers were cut with a diamond pen to obtain an overall length of 60 mm, as illustrated in Fig. 6a.

### Optical characterizations and SEM imaging

The optical transmission spectrum of the AAW_15_ glass was measured between 400–3300 nm by using a UV-VIS-NIR Varian Cary 5000 double beam spectrophotometer on polished 3 mm-thick samples. The optical fiber propagation loss was measured using a fiber cut-back experimental set-up comprising a tungsten-halogen white light source, a xyz stage, a 10x microscope objective from Olympus and an Ando AQ6315A Optical Spectrum Analyzer (OSA). Both cross-sections of the fibers were polished before conducting the measurements. All manipulations have been performed under ambient atmosphere at a temperature of (22 ± 0.5) °C and relative humidity of (45 ± 5) %. The fiber’s surface were imaged by scanning electron microscopy (SEM, FEI, model Quanta 3D FEG).

### Electrical characterizations

The electrical characterizations of the fibers were performed using a 1260 Solartron impedance analyser in the frequency range of 1 Hz to 1 MHz, with an applied voltage of 1 V and zero bias with an accuracy of 0.1%. The 2-point conductivity method was used using a home-made fiber holder with two stainless steel holes electrodes with a 4-terminal measuring device that eliminates lead or parasite resistances on the electrodes. To allow better contact between the stainless steel electrodes and the fibers, silver paint was applied on both contact surfaces of the fiber. The silver paint used was colloidal silver from Pelco with a sheet resistance of 0.02–0.05 Ω/sq/mil and a service temperature range between −40 °C and 260 °C. After application of the silver paint on the fibers’ ends, they were fixed in their holder and the silver paint was dried at 60 °C during 30 minutes to allow an ohmic contact. Electrical conductivity measurements were then performed as a function of temperature, from 25 °C up to 75 °C.

## Additional Information

**How to cite this article**: Rioux, M. *et al*. Optical and electrical characterizations of multifunctional silver phosphate glass and polymers-based optical fibres. *Sci. Rep.* **7**, 43917; doi: 10.1038/srep43917 (2017).

**Publisher's note:** Springer Nature remains neutral with regard to jurisdictional claims in published maps and institutional affiliations.

## Supplementary Material

Supporting Information

## Figures and Tables

**Table 1:** Electrical conductivity of the single-core glass fibers, the two circular-core fiber and the ring-fiber as a function of the surface/volume ratio.

Fiber geometry	Glass composition	Surface/volume ratio	Conductivity (S·cm^−1^)
Single-core 125 μm	A-AW_15_	320	0.43
Single-core 250 μm	A-AW_15_	120	0.14
Two-circular core fiber	A-A-W_12_	320	0.43
Ring-core fiber	A-A-W_12_	222	0.22

Temperature: 25 °C; Applied voltage: 1 V; Frequency: 1MHz, accuracy: 0.1%. Glass labels: AAW_12_: 45AgI-43-AgPO_3_-12WO_3_, AAW_15_: 45AgI-40-AgPO_3_-15WO_3_.
