# Supplementary material for: Optical and electrical characterizations of multifunctional silver phosphate glass and polymer-based optical fibers
Source: Sci Rep. 2017 Mar 3;7:43917. doi: 10.1038/srep43917 (PMC5335562; doi:10.1038/srep43917)
Supplement: Supporting Information [file srep43917-s1.doc]

Supporting information

**Optical and electrical characterizations of multifunctional silver phosphate glass and polymers-based optical fibres**


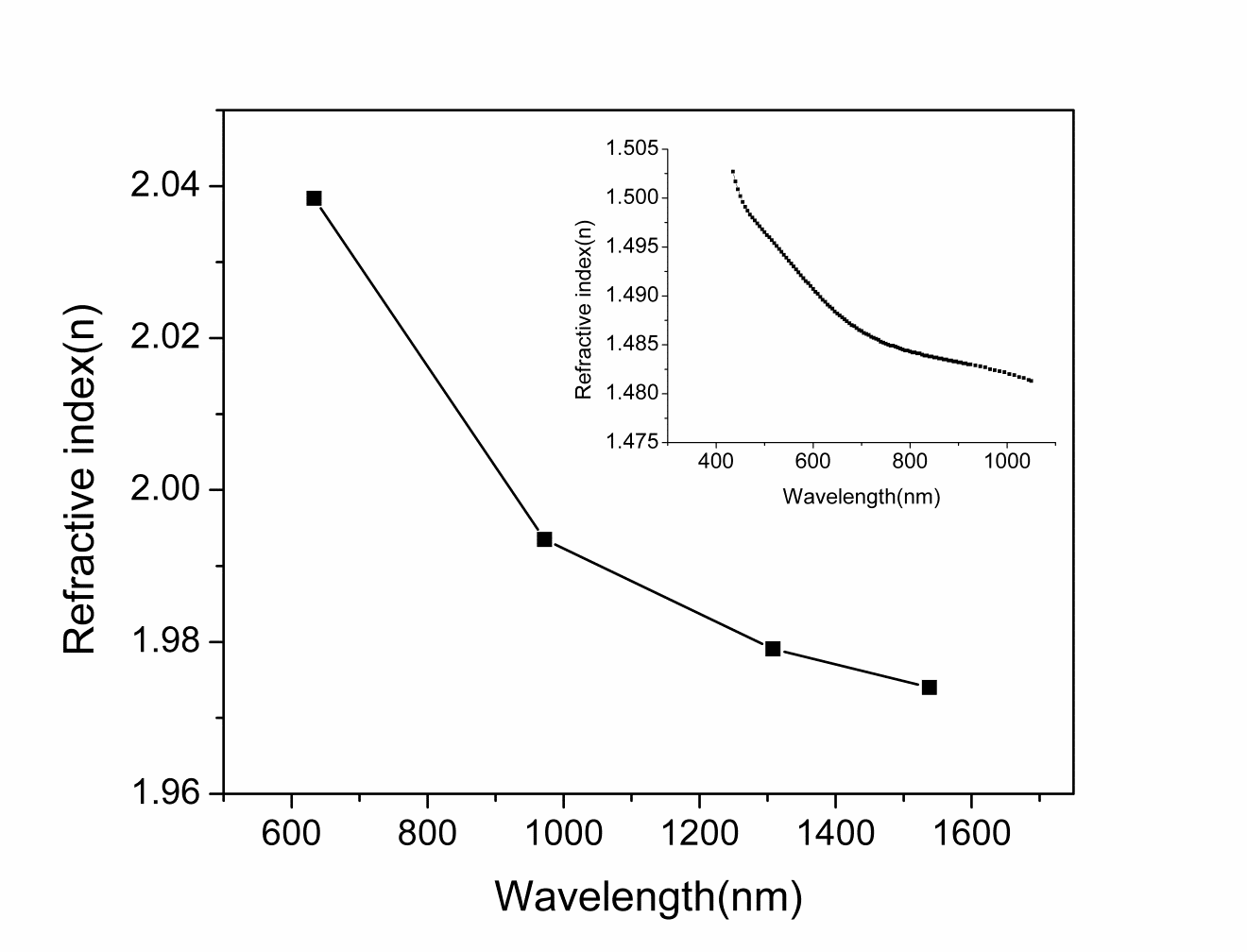
Maxime Rioux*, Yannick Ledemi, Steeve Morency, Elton Soares de Lima Filho, Younès Messaddeq.

**Figure S1**. Refractive index as a function of wavelength for the 45AgI-40AgPO_3_-15WO_3_ (AAW_15_) glass and for acrylic in inset (Plotted from reference [46]).


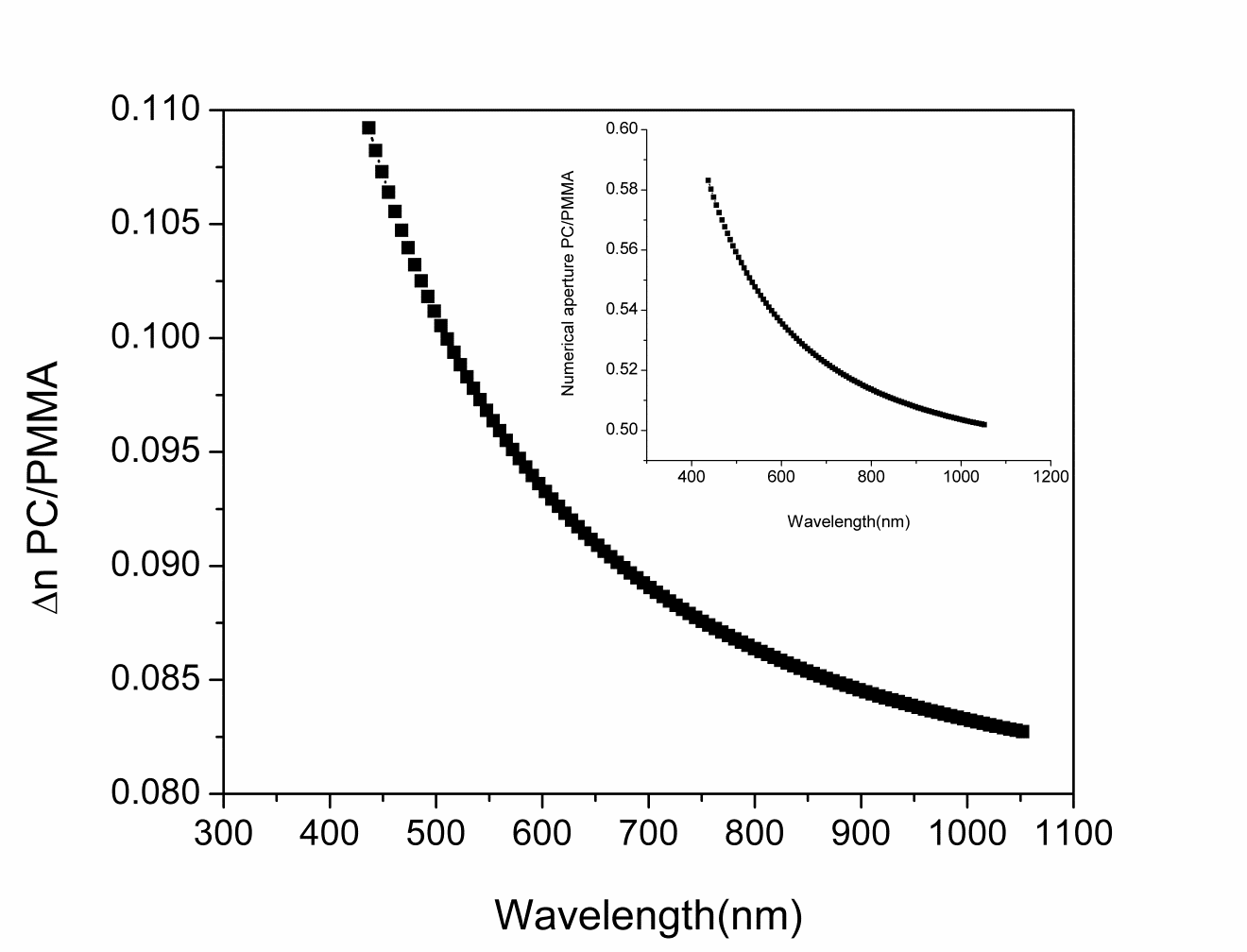


**Figure S2.** Difference in refractive index between polycarbonate (PC) and poly(methyl methacrylate) (PMMA) with the numerical aperture of PC/PMMA core cladding fibre in inset (Both curves are calculated from reference [51]).
